# Development and validation of an HPLC–MS/MS method for the determination of arginine-vasopressin receptor blocker conivaptan in human plasma and rat liver microsomes: application to a metabolic stability study

**DOI:** 10.1186/s13065-018-0414-5

**Published:** 2018-05-02

**Authors:** Haitham Alrabiah, Adnan A. Kadi, Mohamed W. Attwa, Gamal A. E. Mostafa

**Affiliations:** 1 Department of Pharmaceutical Chemistry, College of Pharmacy, King Saud University, P.O. Box 2457, Riyadh, 11451 Saudi Arabia; 2 Micro-analytical Lab, Applied Organic Chemistry Department, National Research Center, Dokki, Cairo, Egypt

**Keywords:** Conivaptan, LC–MS/MS, Human plasma, RLMs, Metabolic stability study

## Abstract

**Purpose:**

To develop and validate a bio-analytical HPLC–MS/MS method for the determination of conivaptan (CVA) an arginine-vasopressin receptor blocker in human plasma and in rat liver microsomes (RLMs).

**Methods:**

Analytes were separated on a reversed phase C18 column (50 mm × 2.1 mm, 1.8 μm). The mobile phase was a mixture of acetonitrile and 10 mM ammonium formate (40:60 v/v, pH 4.0) and was pumped isocratically for 4 min at a flow rate of 0.2 ml/min. Multiple reaction monitoring in positive ionization mode was used for the assay.

**Results:**

The method yielded a linear calibration plot (*r*^*2*^= 0.9977 and 0.9998) over 5–500 ng/ml with a limit of detection at 1.52 and 0.88 ng/ml for human plasma and RLMs, respectively. The reproducibility of detection of CVA in human plasma and RLMs was found to be in an acceptable range.

**Conclusion:**

The method developed in this study is applicable for accurately quantifying CVA in human plasma and rat liver microsomal samples. The optimized procedure was applied to study of metabolic stability of CVA. Conivaptan concentration rapidly decreased in the first 2 min of RLMs incubation and the conversion reached a plateau for the remainder of the incubation period. The in vitro half-life (t_1/2_) was estimated at 11.51 min and the intrinsic clearance (CL_in_) was 13.8 ± 0.48 ml/min/kg.

## Introduction

**Fig. 1:**
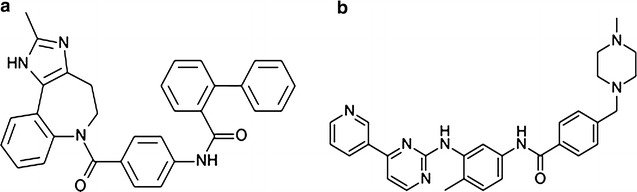
Chemical structure of conivaptan (**a**) and imatinib (**b**)

Conivaptan (YM087, CVA) is a vasopressin receptor antagonist (non-peptide inhibitor of antidiuretic hormone). It is approved for the treatment of hyponatremia [low blood levels of sodium caused by syndrome of inappropriate antidiuretic hormone secretion (SIADH)] [1, 2] under the brand name vaprisol. Its chemical name is “*N*-(4-((4,5-dihydro-2-methylimidazo[4,5-*d*][1]benzazepin-6(1*H*)-yl)carbonyl)phenyl)-(1,1′-biphenyl)-2-carboxamide” (structure shown in Fig. 1).

Vaptans such as CVA and tolvaptan represent a targeted approach to treatment of hyponatremia by inhibiting the interaction of arginine vasopressin with the V2 receptor [2, 3]. Conivaptan inhibits two subtypes of the vasopressin receptors (V1a and V2) and is therefore utilized in the treatment of SIADH. It increases sodium concentration in the blood, and regulates diuresis to prevent water retention in the body [4–7].

Few analytical methods using HPLC-tandem mass spectrometry (HPLC–MS/MS) have been reported as assays of CVA [8, 9], and this technique was previously used for elucidating the pharmacokinetic properties of CVA [8]. However, this method [8] was not fully validated and the separation was carried out using gradient elution with a mobile phase at 40 °C. A second method was reported for screening urine samples for various doping agents using HPLC-high resolution MS. Diuretics including CVA, lixivaptan, mozavaptan, tolvaptan, relcovaptan were screened with this assay [9].

HPLC–MS/MS is an attractive technique because it accurately separates analytes in samples with complex matrices such as biological fluids, containing a variety of environmental contaminants and drugs [10, 11]. It is widely used in bioanalysis, especially pharmacokinetic studies of pharmaceuticals. Pharmacokinetic studies are used to determine the fate of certain drug and how quickly it cleared from the body or a specific organ. Mass detection is useful in these studies because of its very short response time, high sensitivity and selectivity compared with standard chromatographic techniques. Notably, one major advantage of mass detection is that the detector can be tuned to select specific ions to fragment with a very high level of accuracy.

Method validation is required to establish an analytical method that yields accurate, precise, and reproducible results. Reproducibility is an essential requirement for pharmacokinetic, pharmacodynamics, and toxicological studies [8, 12, 13]. Consequently, method validation is a critical step in bio-analytical data collection in drug studies. Hence, all validation parameters should be studied and approved in accordance with Food and Drug Administration (FDA) guidelines on bio-analytical method validation [14].

In this study, we have developed and validated an HPLC–MS/MS method for the detection and quantitation of CVA in human plasma and rat liver microsomes (RLMs). The developed method is completely validated compared with screening qualitative methods [8] and the pharmacokinetic study method (gradient elution at 40 °C) [9]. The proposed method is based on the use of electrospray ionization (ESI) in positive mode as a source of ions and the use of MRM method to detect analytes.

The proposed method was utilized to assess the metabolic stability of CVA by determining its rate of conversion when incubated with RLMs and estimating the associated in vitro half-life (t_1/2_) and intrinsic clearance (CL_in_). Using these and other pharmacokinetic data, such as hepatic clearance (CL_H_), bioavailability and in vitro *t*_1/2_ can be estimated which are very important to aid in defining relationships between in vitro *and* in vivo correlation behavior. Particularly, a common trend is low in vivo bioavailability of compounds that exhibit rapid rates of in vitro metabolism [15].

## Experimental

### Chemicals and reagents

A CVA standard was obtained in powdered form provided by Santa Cruz Biotechnology, Inc. (Heidelberg Germany). Imatinib, used as an internal standard, was obtained from “Sigma-Aldrich (St. Louis, MO, USA)”. Ultra-pure water (18 μΩ) was prepared using a Milli-Q plus purification system (Millipore, USA). HPLC-grade solvent (acetonitrile) was supplied by Merck BDH Ltd. (Poole, UK) product. Ammonium formate and formic acid, analytical grade, were acquired from AVONCHEM (Macclesfield, Cheshire, England). Human blood was a kind donation by “King Khaled University Hospital, King Saud University, Riyadh, Saudi Arabia”. With informed consent acquired from patients, collection of fasted blood samples was carried out followed by separation of plasma, which was kept frozen at − 70 °C. RLMs were prepared and supplied by “the Animal Care Center, Faculty of Pharmacy, King Saud University”. Millex-GP 0.22 µm syringe filters were obtained from Millipore and OMNI homogenizer was supplied by Omni International (Kennesaw, GA, USA).

### Instrumentation and conditions

An Agilent 1200 HPLC system (Agilent Technologies, Palo Alto, CA, USA) in conjunction with an Agilent 6410 triple quadrupole mass spectrometer was used in this study. Elution in isocratic mode was performed using “Agilent Eclipse plus C18 analytical column (50 mm × 2.1 mm, 1.8 μm) maintained at 25 °C”. The mobile phase consisted of acetonitrile and 10 mM ammonium formate (40:60 v/v), pH 4.0, used at a rate of 0.2 ml/min during all experiments. CVA and the internal standard imatinib eluted at 2.780 and 1.293 min, respectively. A total run time and injection volume of 4 min and 5 µl, respectively, were sufficient and appropriate for these experiments. The detector was operated in positive mode with an ESI ion source. Nitrogen was used as a desolvation gas with a flow rate of 12 l/min, and the collision gas was high purity nitrogen at a pressure of 50 psi. A temperature of 350 °C was set for the source and the capillary voltage was set at 4 kV. Quantitation was attained with the aid of MRM target transitions of CVA precursor ion 499.2 → 300.2 and 499.2 → 181.2, in addition to IS precursor 494 → 394.1. Collision energy was set at 25, 12 V for CVA and 20 V for the IS, respectively the dwell time (200 ms) for each ion. CVA was fragmented at 145 and 135 V and the IS was fragmented at 135 V. “Mass Hunter software (Agilent Technologies, CA, USA) was used for operating the instrument and acquiring the data.”

### Preparation of standard solutions

Standard solution of CVA (1000 μg/ml) was freshly prepared in methanol. Imatinib (IS) (1000 μg/ml) stock solution was freshly made in DMSO. Two analyte working solutions at 100 µg/ml (working solution 1) and 10 µg/ml (working solution 2) were prepared in methanol. Two working solution of IS were made by appropriate dilution from stock to give 100 and 2 µg/ml in DMSO. An exact amount was subsequently prepared as dilutions in the optimized mobile phase to make a set of calibration and quality control solutions. All prepared solutions were kept at 4 °C until use.

### RLM sample preparation

Four Sprague–Dawley rats were provided by the Animal Care Center as stated above. Approval of the experimental animal procedure used for preparation of RLMs was previously granted by the Institutional Review Board, King Saud University. Rats were sacrificed by cervical dislocation, and peritoneal cavity incisions were made to harvest the livers. Rat livers were weighed in a clean beaker. A pH 7.4 phosphate buffer solution (consisting of 0.04 M KH_2_PO_4_/NaH_2_PO_4_, 0.25 M sucrose and 0.15 M KCl) was used with rat liver tissue at 1:4 w/v and liver tissue was homogenized using an OMNI homogenizer, followed by centrifugation of the homogenate at 10,000*g* for 22 min at 4 °C. This was followed by centrifugation of the supernatant at 100,000*g* for 70 min and removal of the supernatant. The resultant pellets were then re-constituted in KCl/sucrose buffer and the microsomes were subsequently stored at − 70 °C. The Lowry assay [16] was used to determine its protein concentration. The activity of cytochrome P450 enzymes was quantitated by measuring the bio-activation of phenytoin to *p*-hydroxyphenytoin by the microsomes [17].

### Calibration curve

#### Human plasma

A suitable amount of CVA (10 μg/ml) was diluted in human plasma to obtained eleven concentrations ranging from 5 to 500 ng/ml, with 100 µl of 2 µg/ml IS added to each dilution. Acetonitrile was added to achieve removal of plasma protein. Plasma samples were centrifugation at 10,000 rpm for 20 min at 4 °C. The resulting clear solutions were filtered through 0.22 µm syringe filters then loaded into the auto-sampler and 5 µl of each prepared solution was analyzed by LC–MS/MS. A blank was prepared in a similar manner using human plasma without drug and was injected into the LC–MS/MS to check for interference.

#### Rat liver microsomes

A suitable amount of CVA (10 μg/ml) was diluted into RLMs to yield of eleven samples with concentrations ranging from 5 to 500 ng/ml, then one hundred microliters of 2 µg/ml internal standard was added to each. Acetonitrile was added, and the samples were centrifuge at 14,000 rpm for 12 min at 4 °C. The clear solutions were removed and filtered through 0.22 µm syringe filters. The clear filtrates were placed into the auto-sampler and a volume of 5 µl of each solution was assayed by the LC–MS/MS system. A blank constituting RLMs matrix without the drug was analyzed using the same protocol with the mobile phase rather than RLMs. Blanks were injected into the LC–MS/MS to identify interferences.

Calibration curves (at concentrations 5, 10, 15, 20, 30, 50, 100, 150, 300, 400 and 500 ng/ml) were generated for spiked human plasma and RLMs samples by plotting peak area ratio for CVA to IS on the *y* axis versus CVA nominal concentration levels on the *x* axis. Each data point was tested in six replicates. The parameters of the calibration curve parameters, including the slope of the line of best fit, its intercept, and correlation coefficient (r^2^) values were calculated. CVA concentrations in the spiked RLM samples were computed by substituting their ratios into the generated linear regression equation.

### Method validation

The current methods were validated in accordance with the guidelines recommended by the US Food and Drug Administration (FDA) and the International Conference on Harmonisation (ICH) [18, 19] for analytical procedures and methods, as detailed below.

#### Specificity

Six blank plasma samples were analysed using HPLC–MS/MS after extraction to estimate the specificity of the investigated method. Blanks were separated using optimized chromatographic conditions to check for any peaks eluting at the same times as CVA or IS. Carryover effects were tested by increasing the elution time of separation and raising post run time to check for any other peaks which may interfere with drug detection. Moreover, MRM spectra of blanks with mass fragmentation patterns of CVA and IS were obtained to check the specificity of the method.

#### Extraction and matrix effects

Different methods of extraction were tested using ethyl acetate liquid–liquid extraction, solid phase extraction, and protein precipitation. Protein precipitation using acetonitrile as the protein precipitating solvent was proven to be the best method, in which show more than 94% recovery was attained. An extract sample was also spiked with a known concentration of CVA and its percentage recovery was compared with analyte sample spiked into mobile phase. The recovery percentage was approximately 98.0%.

#### Linearity and sensitivity

Assessment of the linearity of the developed method was carried out using six different calibration curves, which were plotted based on peak area ratios of CVA to the internal standard imatinib on the *y*-axis in relation to the assayed concentrations of CVA on the *x*-axis. Briefly, 11 concentrations of calibration solutions (5–500 ng/ml) were prepared fresh every day by spiking CVA into human plasma samples. Data generated for calibration were analysed by least-squares linear regression to establish the range of linearity. Assessment of the sensitivity of the assay was performed following ICH recommendations [18], by estimating the limits of detection (*LOD*) and quantitation (*LOQ*) of the technique using the slope of the constructed calibration line and the standard deviation associated with its intercept based on the equation below.

$$LOD\;or\;LOQ = k \frac{{SD_{b} }}{a}$$

where *k* equals 3 and 10 for *LOD* and *LOQ*, respectively, SD_b_ represents the standard deviation associated with the intercept, and *a* denotes the slope of the plot.

#### Precision and accuracy

Determinations of intra-day precision and accuracy were carried out via analysis of spiked human plasma and RLMs using three QC samples which were estimated from previous calibration curves. Their values were estimated during a day and on different days. Precision was expressed as $\% RSD = \left[ {\left( {{{SD} \mathord{\left/ {\vphantom {{SD} {Mean}}} \right. \kern-0pt} {Mean}}} \right) \times 100} \right],$ whereas accuracy was assessed as % relative error or % recovery: $\% RE = \left[ {{{\left( {Conc_{measured} - Conc_{nominal} } \right) } \mathord{\left/ {\vphantom {{\left( {Conc_{measured} - Conc_{nominal} } \right) } { Conc_{nominal} }}} \right. \kern-0pt} { Conc_{nominal} }}} \right] \; \times \;100.$

#### Stability

Stability of conivaptan in human plasma and RLM samples by analyzing QC samples in six replicates assessed in several storage conditions relevant to routine sample processing. Measurements of mean CVA concentrations, accuracy and precision values were calculated based on freshly constructed plasma calibration curves. Stability of CVA was assessed by incubating QC samples at room temperature for 8 h, storing samples at 4 °C for 24 h and storing samples at − 20 °C for 30 days. Freeze–thaw stability was assessed using three cycles carried out by freezing at − 70 °C and then thawing at 25 °C.

#### Sample integrity and incurred sample

A stock solution of CVA at 1.8% higher CVA concentration than highest concentration standard in the calibration range was prepared in methanol. Two diluted concentrations (90 and 45%) were prepared in spiked human plasma and RLMs. The concentration of CVA in human and RLMs were determined from the previously prepared calibration curve. Three QC samples of spiked human plasma and RLMs were assessed a second time after 7 days for incurred sample reassessment.

### Method application

#### Assessing the metabolic stability of CVA

This metabolic stability study was designed to track the disappearance of CVA incubated with RLMs by measurements of the drug based on the developed LC–MS/MS assay. Tests were carried out in three replicates at a final concentration of 1 µM CVA in 1 mg/mL microsomal protein, with 1 mM NADPH and 3.3 mM MgCl_2_ in phosphate buffer (pH 7.4) in 1 mL total volume. NADPH was used to initiate the incubation reaction and 2 mL of acetonitrile was used to terminate it at different times ranging from 0.0 to 50.0 min. Solvent-precipitated proteins were then isolated by centrifugation at 10,000 rpm for 17 min at 4 °C and the resultant clear solution was filtered using 0.22 µm syringe filters and IS (100 µl) was added to 1 mL of the filtered supernatant. Five microliters of the filtrate was assessed by LC–MS/MS. Finally, the concentration levels of CVA in the incubations were estimated using the pre-calibration plot of CVA in RLMs.

## Results and discussion

### Chromatographic conditions and MS

Optimization of the chromatographic and mass spectrometric methods and experimental conditions to enhance the resolution and sensitivity of the assay and obtain the highest quality mass response were achieved after several re-assessments. Because the pH level of the aqueous solution in the mobile phase will determine the degree of ionization of dissolved compounds, produce ion suppression or enhancement effects, and modify the shape of analyte peaks, different mobile phase compositions were assessed.

Formic acid was tested at 0.1% with different ratios of acetonitrile; the pH of these mobile phases was 3.1. This mobile phase produced peak separation with slight tailing. We also used ammonium formate at pH 4.0 in combination with acetonitrile. This mobile phase offered good quality peak separation. Drug and IS peaks were well separated and there was no peak tailing because at pH 4.0 the drug was present completely in one ionic form, pKa 6.23 [20]. Therefore, we changed the mobile phase from formic acid to ammonium formate to increase the pH of the mobile phase. Two different concentrations of ammonium formate buffer were tested (5 mM and 10 mM) for their effect on separation, resolution and peak symmetry. Ammonium formate at 10 mM resulted in better chromatography than 5 mM. Therefore, ammonium formate:acetonitrile (40:60 v/v, pH 4.0) was used at 0.2 ml/min flow rate in isocratic mode.

Different drugs were tested for use as the internal standards. The choice of internal standard was based on it having similar chemical properties to those of the target analyte to be separated and on its absence in the endogenous sample to be analyzed. Imatinib has the same functional groups, a similar boiling point, and a similar pKa [21] to those of CVA. Therefore, in this investigation we used imatinib as an internal standard, which can be separated under optimized conditions from CVA.

**Fig. 2:**
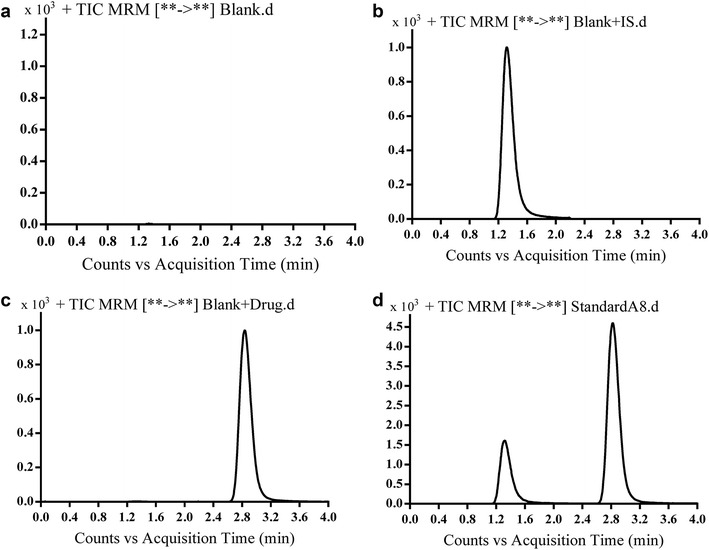
Total ion chromatogram of MRM of **a** blank, **b** blank spiked with 200 ng/ml of IS, **c** blank spiked with 100 ng/ml of CVA and **d** blank spiked with 200 ng/ml IS and 100 ng/ml drug

Under optimized conditions, CVA and the IS eluted at 2.780 and 1.293 min, respectively under the recommended LC–MS/MS conditions. Complete chromatographic elution of both CVA and IS was achieved within 4 min (Fig. 2). The system suitability parameters of CVA were 26.8, 2.246 and 1.9 for capacity factor, separation factor, and resolution, respectively. The tailing factor was about 1 for both CVA and IS. These data indicate that the separation criteria are in accordance with FDA guidelines [19]. Peaks of conivaptan and IS peaks showed high resolution, with no evidence of carryover into blank samples or CVA-free QC solutions (blanks with spiked IS). Figure 2 shows representative chromatograms of 100 ng/ml CVA, internal standard and blank samples.

**Fig. 3:**
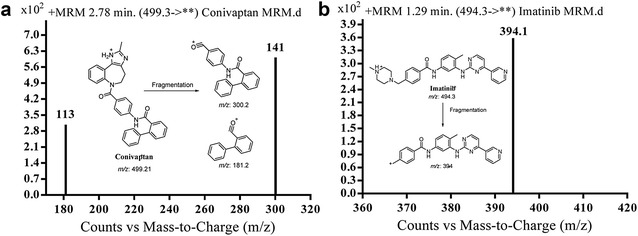
MRM mass spectra of conivaptan (**a**) and IS (**b**) and the supposed fragmentation path way

In a similar manner, mass detection parameters were improved in order to increase the ionization efficiency of the drug and internal standard precursor and main fragment ions. Minimization of likely interfering peaks and improvement of the sensitivity of the system were accomplished by means of the MRM mode. To obtain the best sensitivity, ESI was operated in positive mode for HPLC–MS/MS analysis. Product ions of CVA (at *m/z* 499.2) were mainly ions at [M+H]^+^ *m/z* 300.2 and 181.2. The product ion of IS ion (*m/z* 494.1) was one significant ion at [M + H]^+^ *m/z* 394. These transitions were selected to be monitored in the MRM mode of analysis of CVA and IS in order to provide optimized quantitation of the analyte with adequate sensitivity and selectivity (Fig. 3).

### Method validation

#### Specificity

The specificity of the developed assay is indicated by the absence of peaks at CVA and/or IS retention times in analyzed blank solutions. Moreover, carryover was not observed in the analyzed samples. Separation of CVA and IS was achieved using optimum HPLC conditions with elution times of 2.780 and 1.293, respectively. Moreover, MRM of the bank was recorded with fragmentation targeted at the masses of the investigated drug and IS. The intensity of the blank at these masses was approximately zero, representing noise peaks with no evidence of positive detection of analyte.

#### Linearity and sensitivity

The developed method was shown to be robust and sufficiently sensitive for day-to-day analysis of CVA in laboratory and clinical settings. RSD values estimated based on linear regression for a range of CVA concentrations were shown to be within 4.15%. A linear response was illustrated for a calibration range of 5–500 ng/ml, with a regression equation *y *= 0.9882*x* + 3.8301 and y = 1.6593*x* + 0.8199, and correlation coefficient *r*^*2*^ of 0.9977 and 0.9998, for human plasma and RLMs, respectively. The LOD and LOQ were (1.52 and 5 ng/ml) and (0.88 and 5 ng/ml) respectively, which allows easy detection of CVA in RLMs (results shown in Table 1). To demonstrate optimal function of the investigated assay, QC plasma samples were assessed to determine the back-calculated levels of CVA. CVA quantitation accuracy and precision in back-calculated samples were in the range 97.54–101.65 and 0.68–4.15%, respectively.

**Table 1:** Back calculation of conivaptan

Conc. (ng/ml)	Human plasma					RLMs				
	Mean	SD	RSD	R (%)	RE (%)	Mean	SD	RSD	R (%)	RE (%)
5.00	4.95	0.15	2.99	98.90	− 1.10	5.05	0.12	2.43	100.95	0.95
10.00	9.95	0.41	4.15	99.54	− 0.46	10.17	0.37	3.69	101.65	1.65
20.00	19.55	0.53	2.73	97.73	− 2.27	19.96	0.35	1.74	99.79	− 0.21
30.00	29.49	0.83	2.81	98.30	− 1.70	30.11	0.67	2.23	100.38	0.38
50.00	49.24	1.09	2.21	98.47	− 1.53	50.28	0.56	1.12	100.56	0.56
100.00	97.88	1.40	1.43	97.88	− 2.12	99.97	1.28	1.28	99.97	− 0.03
300.00	292.62	4.16	1.42	97.54	− 2.46	298.84	1.67	0.56	99.61	− 0.39
500.00	489.50	8.79	1.80	97.90	− 2.10	499.89	3.39	0.68	99.98	− 0.02

#### Precision and accuracy

The level of reproducibility of the developed assay was examined by measuring intra- and inter-day precision and accuracy of CVA quantification using QC samples. The levels of accuracy were reported as % relative error and determined by the formula described in the Experimental section. Precision was reported as intra- and inter-day % RSD measured as described above. Table 2 shows a summary of accuracy and precision testing data, which demonstrate that shows that these parameters are within acceptable standards in accordance with ICH guidelines [18, 19].

**Table 2:** Intra- and inter-day precision and accuracy of quality control sample

Parameter	Human plasma			RLMs		
	LQC (15 ng)	MQC (150 ng)	HQC (400 ng)	LQC (15 ng)	MQC (150 ng)	HQC (400 ng)
	Intra-day					
Mean	14.75	147.11	392.75	15.01	149.65	399.54
SD	0.47	2.34	6.22	0.43	1.91	5.16
RSD (%)	3.18	1.59	1.58	2.90	1.28	1.29
RE (%)	− 1.64	− 1.93	− 1.81	0.05	− 0.23	− 0.12
	Inter-day					
Mean	14.74	146.99	392.28	14.98	149.40	398.72
SD	0.44	2.82	7.36	0.37	2.20	5.90
RSD (%)	3.01	1.92	1.88	2.49	1.47	1.48
RE (%)	− 1.74	− 2.01	− 1.93	99.86	99.60	99.68
Accuracy	98.26	97.99	98.07	− 0.14	− 0.40	− 0.32

#### Stability

**Fig. 4:**
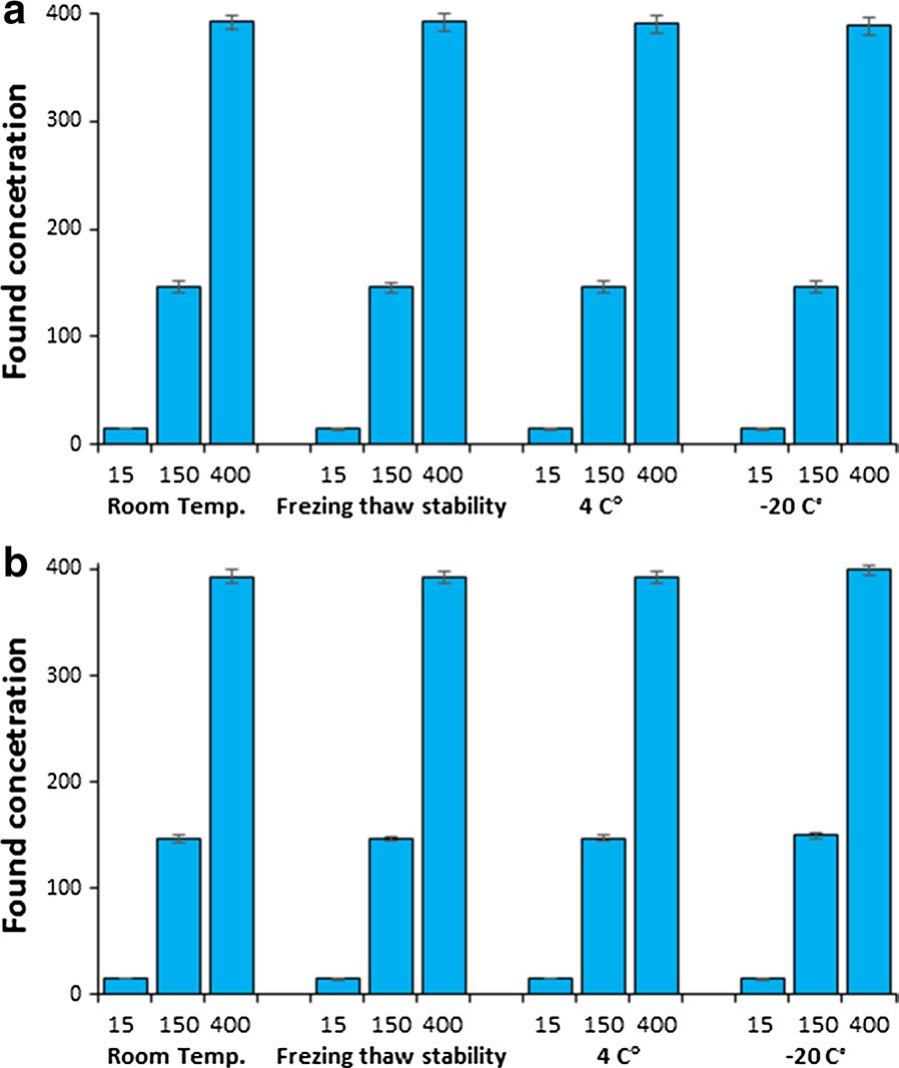
Conivaptan stability data in human plasma (**a**) and in RLMs (**b**) under different conditions, x-axis is the tested concentrations and y-axis is found concentrations (mean ± SD)

Stability assessment was carried out based on CVA QC samples under several storage conditions. Assayed QC samples returned measurements which differed from mean response of fresh samples by ≤ 4.5%. There was no significant degradation observed as a result of storage or handling conditions under study. Results of stability testing (Fig. 4) suggest that no loss of CVA may occur when handling human plasma or RLMs samples under normal laboratory conditions. Incurred QC samples were re-assessed after 7 days. Results of stability tests are presented in Table 3.

**Table 3:** Dilution integrity and incurred samples

	Dilution integrity				Incurred samples					
	Human plasma		RLMS		Human plasma			RMLs		
	Conc. (ng/ml)		Conc. (ng/ml)		Conc. (ng/ml)			Conc. (ng/ml)		
	450	225	450	225	15	150	400	15	150	400
Mean	431.1	221.35	438	221.5	14.6	146.6	391.1	14.77	147.2	393.1
SD	16.24	3.74	2.50	1.85	0.39	2.82	7.17	0.45	1.89	4.77
RSD (%)	3.76	1.68	10.9	4.105	2.65	1.92	1.83	3.02	1.28	1.21
R (%)	95.8	98.37	97.3	98.4	99.3	101.8	100.7	98.44	98.17	98.28
RE (%)	− 4.2	1.63	− 2.6	1.52	− 6.5	1.82	0.75	1.57	− 3.73	− 3.28

n = 6

#### Metabolic stability study

**Fig. 5:**
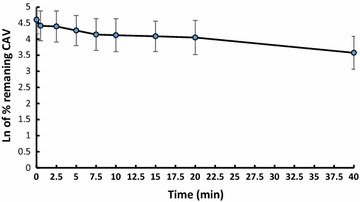
The metabolic stability profile of conivaptan after incubation with RLMs. Metabolic reaction was stopped at different time points (mean ± SD)

Drug metabolic stability tests are conducted to determine the rate of decrease in drug levels within a certain testing system. This approach is justified as a reproducible, simple and cheap in vitro metabolic stability study that can help predict in vivo hepatic clearance resulting from metabolism [22]. Figure 5 shows the microsomal stability of CVA by quantifying its presence after different incubation periods. The metabolic stability was reported as drug in vitro half-life (t_1/2_) at 11.51 min [23] and intrinsic clearance (CL_in_) [24] at 13.8 ± 0.48 ml/min/kg.

## Conclusions

A bio-analytical LC–MS/MS method for the quantification of CVA in human plasma and RLMs was developed, optimized and validated. Linearity was demonstrated for the proposed method over the range of 5–500 ng/ml. Accuracy and prevision of CVA analysis was confirmed in both intra- and inter-day settings, with high levels of recovery from human plasma and RLMs. Conivaptan was shown to be stable in different samples and under several tested laboratory processing and storage conditions. The optimized method was successfully applied to estimate CVA metabolic stability in RLMs. In conclusion, the developed LC–MS/MS method can be instrumental in assessing CVA pharmacokinetics in routine clinical drug monitoring even at low plasma concentrations. The method can likewise be utilized to examine the metabolic profile of CVA in different biological samples.
